# Crossing the rice-wheat border: Not all intra-cultural adaptation is equal

**DOI:** 10.1371/journal.pone.0236326

**Published:** 2020-08-21

**Authors:** Alexander S. English, Nicolas Geeraert

**Affiliations:** 1 Intercultural Institute, Shanghai International Studies University, Shanghai, China; 2 Department of Psychology, University of Essex, Colchester, United Kingdom; Universitat de Valencia, SPAIN

## Abstract

This study aimed to test whether or not where people come from and move to impacts their method for dealing with stress. We investigated this research question among newcomers crossing between the rice and wheat farming regions in China—south and north China, respectively. New evidence suggests wheat-farming agriculture fosters a coping strategy of changing the environment (primary coping), while rice-farming regions foster the converse strategy of fitting into the environment (secondary coping). Using two longitudinal studies on newcomers at universities located in both the rice and wheat farming regions, we hypothesized that students from south China (rice region) at a university in north China (wheat region) would use more primary coping and it would lead to better adaptation (Study 1). In contrast, students from wheat-farming regions moving to a rice university would benefit from secondary coping as an effective strategy for buffering stress (Study 2). Results indicated that for students from rice-farming regions who were studying universities in wheat-farming regions, secondary coping was damaging and attenuated the stress-adaptation relationship. However, in study 2, the reverse was found, as secondary coping was found to buffer the negative effects of stress on sociocultural adaptation for students from wheat-farming regions who were studying at universities in rice-farming regions. This study lends further support to the theory that ecological factors impact how individuals cope with the acculturative stress of moving to a new environment.

## Introduction

Around 258 million people worldwide currently reside outside their country of birth. However, this number is overshadowed by an estimated 700 million people who migrate within their birth country’s borders [1]. Although mobility is accelerating—particularly due to urbanization in Africa and Asia [2]—internal migration has to date received little attention from social psychologists. Recently, scholars s have highlighted some important cultural differences within cultures [3, 4], which would suggest that *intra*-cultural travel, like its inter-cultural counterpart, requires similar adaptation to the novel cultural environment. Across two studies, we test the idea that within-country cultural differences will moderate the utility or effectiveness of different adaptation styles or strategies used by Chinese university students.

### Intra-cultural differences in China

Before it was ever investigated in China, the relationship between culture and ecology had received the attention of scholars in other contexts. In the 1970s, some instrumental work in Canada among indigenous groups showed that different ecological engagements, subsistence strategies, social and cultural structures, and socialization practices fostered different social and cognitive abilities [5]. Similarly, in the Central African Republic, Biaka hunters [‘Pygmies’] and Bagandu farmers who engaged the forest differentially had the predicted different psychological outcomes [6]. Other ecological and psychological processes have found differences within countries like Japan [7], the USA [8], Turkey [9] and India [10, 11].

In China, behavioral differences have been demonstrated both across the urban-rural divide [12], and economic regions [13, 14]. In addition, ecological variability within China has been associated with psychological differences in cognitive thinking [4], collectivistic orientation [15] and interpersonal communication [16].

Talhelm and colleagues [4] found evidence that thinking style in China varied based on the types of farming carried out in different regions. Rice-paddy farming (in the south) requires farmers to coordinate shared irrigation networks and labor exchange, which may have given southern China a tighter, more interdependent culture. In contrast, wheat farming in the north is characterized by independence and autonomy, as crops can be managed on a more individual level. People originating from rice regions have been demonstrated to think more holistically and show more social interdependence than people from wheat regions [4]. Despite a plethora of research on this topic, the impact of such ecology on internal migration has yet to be investigated.

### Migration, stress, coping, and adaptation

It has been well-established that the transition to a new cultural environment, whether abroad or within one’s home country, can cause acculturative stress for migrants, and that this stress is most easily observed in its effect on psychological and socio-cultural adaptation [17]. Both the physical (e.g. climate) and social environment (e.g. cultural values, social norms or thinking) can be extremely challenging for newcomers [18]. For instance, scholars have carried out some investigations on how these differences in physical and social environment directly impact international sojourners [19]. While some studies have similarly investigated the impact of such stressors on the migrant working population in China, there are still far too few for scholars to consider this population represented [13].

To date, the transactional model of coping has been extremely powerful in describing how people deal with stressful situations, such as cross-cultural migration [20, 21]. Coping is conceptualized as the interaction of the individual with their environment when they encounter challenges, which, in the case of migrants, are brought on by their new cultural context [22]. Some evidence has shown that coping strategies themselves are not disconnected from cultural conditioning processes; rather, different cultures normalize different coping strategies. For migrants, a crucial concern is that the coping strategy which they employ may not be appropriate or acceptable in their new environment [23].

One powerful example of this can be seen in well-researched primary and secondary coping strategies. People using primary coping try to directly alter the external environment through action or behavior modification to reduce stress. People in independent cultures are more likely to use primary coping [24]. In contrast, people using secondary coping try to alter either themselves or their appraisal of the situation. Secondary coping is more common in interdependent cultures [25].

Interestingly, one particular coping style is not generally more effective than another in all circumstances [26]. Instead, coping strategies are most effective when the individual’s coping strategy is congruent with the prevailing norm of their surrounding culture [27]. Among international students, for example, matched coping styles have been shown to be more effective than mismatched coping styles on adaptation outcomes [28, 29]. However, this finding has not yet been tested, nor replicated, in the context of internal mobility.

### Rationale of study & hypotheses

This research advances the theoretical proposition that *within-country* ecological differences will influence the capacity for internal migrants to cope effectively with their new cultural environment. This study takes Chinese migrant students as a population of interest—in particular, those who migrate between the rice and wheat-farming regions in China. As research has shown that these two regions align with interdependent and independent cultures, respectively, it follows that students from these regions are culturally conditioned to use either primary or secondary coping when encountering challenges. Thus, building upon the coping congruence hypothesis [23]we examine the effectiveness of coping styles used among a sample of students from both the rice-farming (interdependent) and wheat-farming (independent) regions of China, who have migrated to the alternate region to attend university.

Differences in self-construal have been linked with coping styles [30]. Because individuals from independent cultures are encouraged to explore and control their environment, they would be expected to rely more on primary coping, in which people pursue their private goals and personally solve their problems. Thus, primary coping would be a culturally appropriate coping strategy in the wheat-farming regions, but secondary coping would not. In sharp contrast, in interdependent cultures connections between individuals are emphasized. People are required to align themselves with others within their social environment; thus, secondary coping would be a preferred strategy. Therefore, secondary coping would be more adaptive in the rice-farming regions, but primary coping would not.

This research examines the effectiveness of primary coping at an *independent* site, a university in the northern wheat region of China (Study 1) and that of secondary coping at an *interdependent* site, a university in the southern rice region of China (Study 2). These two sites are relatively equal prestigious STEM institutions and located in a similar population dense area. This equality allows us to weed out differences due to site effects. We therefore can hypothesize that adaptation will be a function of both migrants’ culture of origin, and culture of destination. Adaptation is expected to be strongest when origin and destination culture are congruent (rice-to-rice or wheat-to-wheat move), and weakest when these cultures are incongruent (rice-to-wheat or wheat-to-rice move).

### Study 1

A hierarchical regression tested the predictions of within-culture group variation based on origin of agriculture region and the effectiveness of coping to reduce stress on arrival in a novel cultural context. It was expected that due to crossing the rice/wheat border, the rice ‘away’ group would experience lower socio-cultural adaptation time 2 and would find that their coping strategies function differently from the wheat ‘home’ group. To test our predictions on the utility of coping strategies, we further expected variation in coping (primary vs. secondary strategies) based on origin of group.

## Method

### Participants

**Fig 1:**
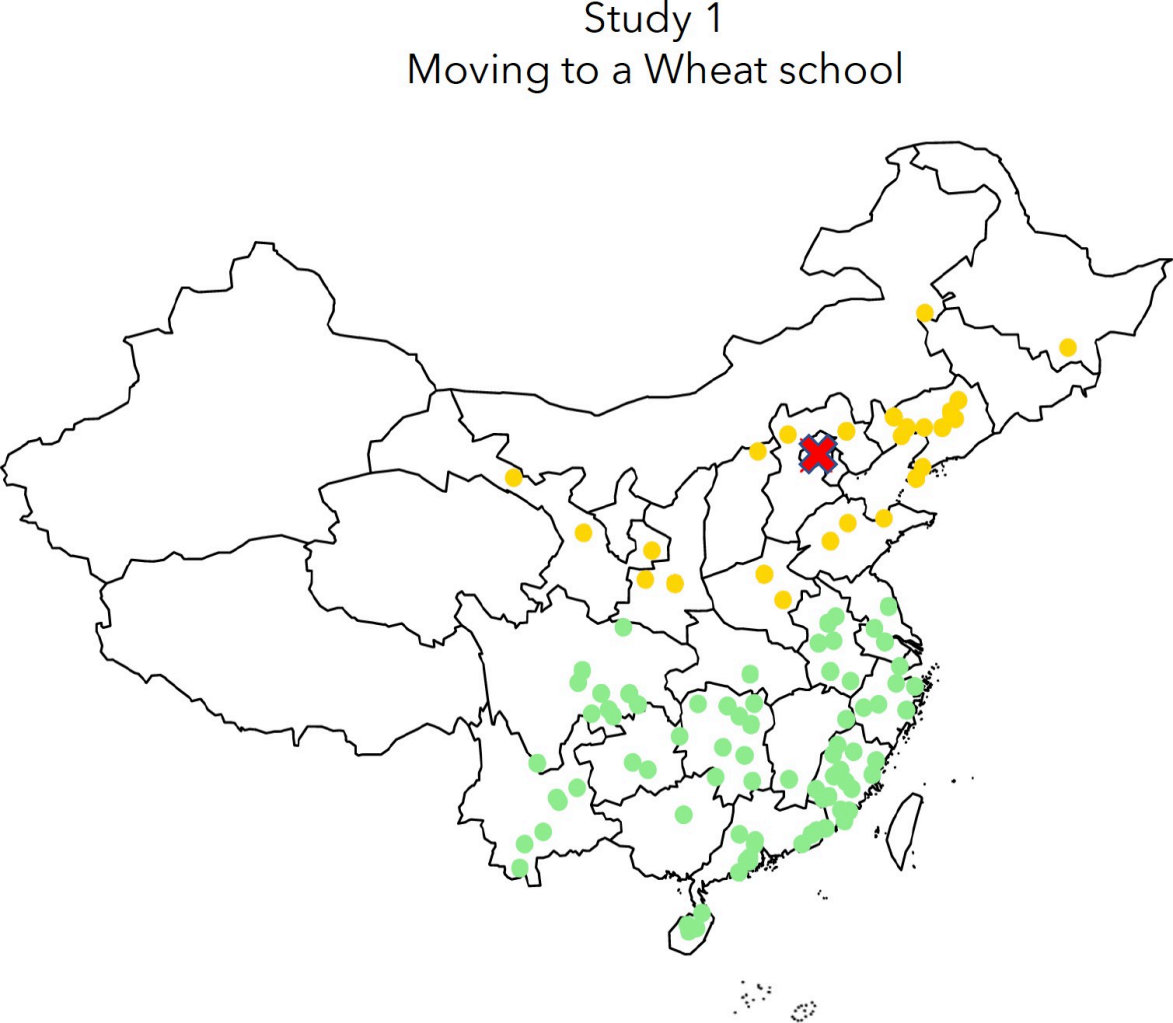
Location of student’s hometown in Study 1. Participants origin were recorded based on their hometown. Yellow dots represent where students from wheat areas originated from. Green dots represent where students from rice areas originated from. Test site was a top-tier university in Beijing.

We tested 198 first-year students originating from wheat (*N* = 90) and rice (*N* = 108) regions in China studying at a university in the northern wheat region (Beijing) at two time points. Participants originated from various parts of China (Fig 1).

### Procedure

Participants were recruited at the start of their first semester, at which point they completed the first survey. A self-generated code was used to match participants between time waves. In line with other studies, we conducted a three-month longitudinal design, first measuring acculturative stress, primary and secondary coping strategies, and perceived cultural distance [31, 32], and adaptation in the second time wave. All procedures and measures were approved by the recommendations of the Institutional Review Board of the Psychology Department of the University with written informed consent from all participants. All subjects gave written informed consent in accordance with the Declaration of Helsinki.

### Materials

All instruments were back-translated by two English-Chinese bilinguals. Time 1 variables included perceived cultural distance, perceived stress, primary coping, and secondary coping. The Time 2 variable was sociocultural adaptation.

#### Perceived cultural distance

The Brief Perceived Cultural Distance scale was used to assess participant’s subjective cultural distance between their homes and the present locations [33]. Students were asked to compare their homes to their current city (i.e. “*How similar or different do you find living in this city*?”) on 12 items that scaled from 1 (*very different*) to 7 (*very similar*). The scale was reverse coded such that higher scores indicate more cultural distance. The scale had good reliability (α = .88).

#### Acculturative stress

Participants completed a brief version of the perceived stress scale [34]. This scale is commonly used in acculturation research [18]. Participants were asked on a scale of 1 (*never*) to 7 (*always*), “In the last 2 weeks how often have you felt…” which was followed by 7 items, such as “felt nervous or stressed”. The scale had good reliability (α = .82).

#### Coping strategies

The COPE Inventory was used to assess coping strategies [35]. Participants were instructed to “Think about any difficult times you have experienced in the last two weeks and what types of strategies are you presently using to overcome these difficulties?”. For each method listed, participants would respond on a scale from 1 (*do not do this at all*) and 4 (*do this a lot*).

Primary coping (α = .82) consisted of eight items, such as “I concentrate my efforts on doing something about it”, measuring planning (four items) and active coping (four items). The secondary coping scale (α = .75) consisted of eight items, such as “I accept that this has happened and that it can’t be changed,” measuring positive growth and reinterpretation (four items) and acceptance (four items). Both constructs have been implemented effectively in various cross-cultural adaptation studies (25; and for implementation, 31; 32).

#### Adaptation

The Brief Sociocultural Adaptation Scale (33) assessed the ease of adapting, or behaviorally “fitting in”, to the social and cultural context of the student’s university’s host city. Participants were asked to indicate, “How easy or difficult do you think it is to adapt to. . .” items such as: social norms, population density, climate, and food, using a 12-item scale anchored from 1 (*very difficult*) to 7 (*very easy*). Reliability was good (α = .92).

#### Preliminary analyses

The participants originating from rice and wheat cultures were not significantly different with regards to gender (65% female, χ^2^ = 2.85, *p* = .09) or the time individuals had already been living in the city (*t* < 1). However, there was a slight difference in age, *t* (196) *=* -3.48, *p* = .001, indicating that participants from rice areas (later called ‘rice group’) were slightly older (*M* = 19.89 *SD* = 2.15) than participants from wheat-farming regions (later called ‘wheat-group’) (*M* = 19.01, *SD* = 1.14). A post-hoc power analysis was conducted and found this sample size has a power of .80 to detect a medium effect size. This is in line with other rice-wheat studies [36].

In term of attrition, we analyzed whether participants who completed both timewaves (70%) were different from those who dropped out. Importantly, analyses indicated that attrition participants were not different with regards to stress (*t* < 1), region of origin (*t* < 1), or time living in the city (*t* < 1). However, compared to retained participants, those who dropped out were slightly older (*t* = 2.47, *p* = .015), perceived more cultural distance (*t* = -3.55, *p* = .001) and endorsed both primary (*t* = 2.45, *p* = .015) and secondary coping more (*t* = 1.97, *p* = .05).

## Results

### Longitudinal moderation analysis

To examine whether the relationship between stress and adaptation was moderated by variations in coping and student geographical origin, a four-step hierarchical regression was conducted (Table 1). In the first step, sociocultural adaptation was regressed on control variables. The second step added stress (at t1), primary coping (at t1), secondary coping (at t1), and students’ rice/wheat origin. The addition of these variables improved the model significantly, Δ*F* (4, 181) = 4.88, p < .001. Origin (rice vs. wheat), primary coping, and secondary coping emerged as significant predictors. On average, students from the rice region had lower levels of adaptation (*β* = -.19, *p* = .011). Primary coping was (marginally) associated with lower adaptation (*β* = -.15, *p* = .073), and secondary coping with higher adaptation (*β* = .28, *p* = .001). The addition of the two-way interactions, in the third step, did not significantly change the model.

**Table 1:** Regression analyses of stress, origin, and coping predictors on sociocultural adaptation (Study 1).

	step 1 basic model		step 2 main effects		step 3 2-way interactions		step 4 3-way interactions	
	beta	p	beta	p	beta	p	beta	p
cultural distance (t1)	-.37	.00	-.28	.00	-.27	.00	-.26	.00
living time	.05	.51	.05	.43	.06	.41	.06	.38
first time	.03	.69	.03	.64	.03	.66	.02	.72
stress (t1)			-.10	.13	-.10	.27	.03	.75
origin: rice vs wheat			-.19	.01	-.19	.01	-.23	.00
stress x origin					.00	.98	.01	.92
primary coping								
primary coping (t1)			-.15	.07	-.35	.01	-.40	.00
stress x primary					.17	.14	.18	.22
origin x primary					.21	.08	.26	.03
stress x origin x primary							-.10	.36
secondary coping								
secondary coping (t1)			.28	.00	.42	.00	.44	.00
stress x secondary					-.08	.48	.10	.49
origin x secondary					-.16	.12	-.15	.12
stress x origin x secondary							-.23	.05
model statistics								
F (df)	9.96*** (3,185)		7.41*** (7, 181)		4.76*** (12, 176)		5.20*** (14, 174)	
R^2^	.14		.22		.25		.30	
ΔF (df)			4.88*** (4, 181)		1.03 (5, 176)		6.18** (2, 174)	
ΔR^2^			.08		.02		.05	

Categorical Variable: First time to leave hometown (1 = yes; 0 = No) Origin: 0 = Rice hometown, 1 = Wheat hometown.

* *p* <. 05

** *p* < .01

*** *p* < .001

In the final step, two 3-way interactions of stress by coping by origin (one for each coping type) added 5% of unique variance in sociocultural adaptation, Δ*F*(2, 174) = 6.18, *p* = .003. Although the 3-way interaction of stress by origin by primary coping was not significant, the 2-way interaction of origin by primary coping was (*β* = .26, *p* = .025). Simple slope analysis indicated that primary coping was negatively related to sociocultural adaptation for the wheat ‘home’ group (*p* < .01), but not for rice ‘away’ group (*t* < 1). More interestingly, the 3-way interaction of stress by secondary coping by origin was marginally significant (*β* = -.23, *p* = .052).

**Fig 2:**
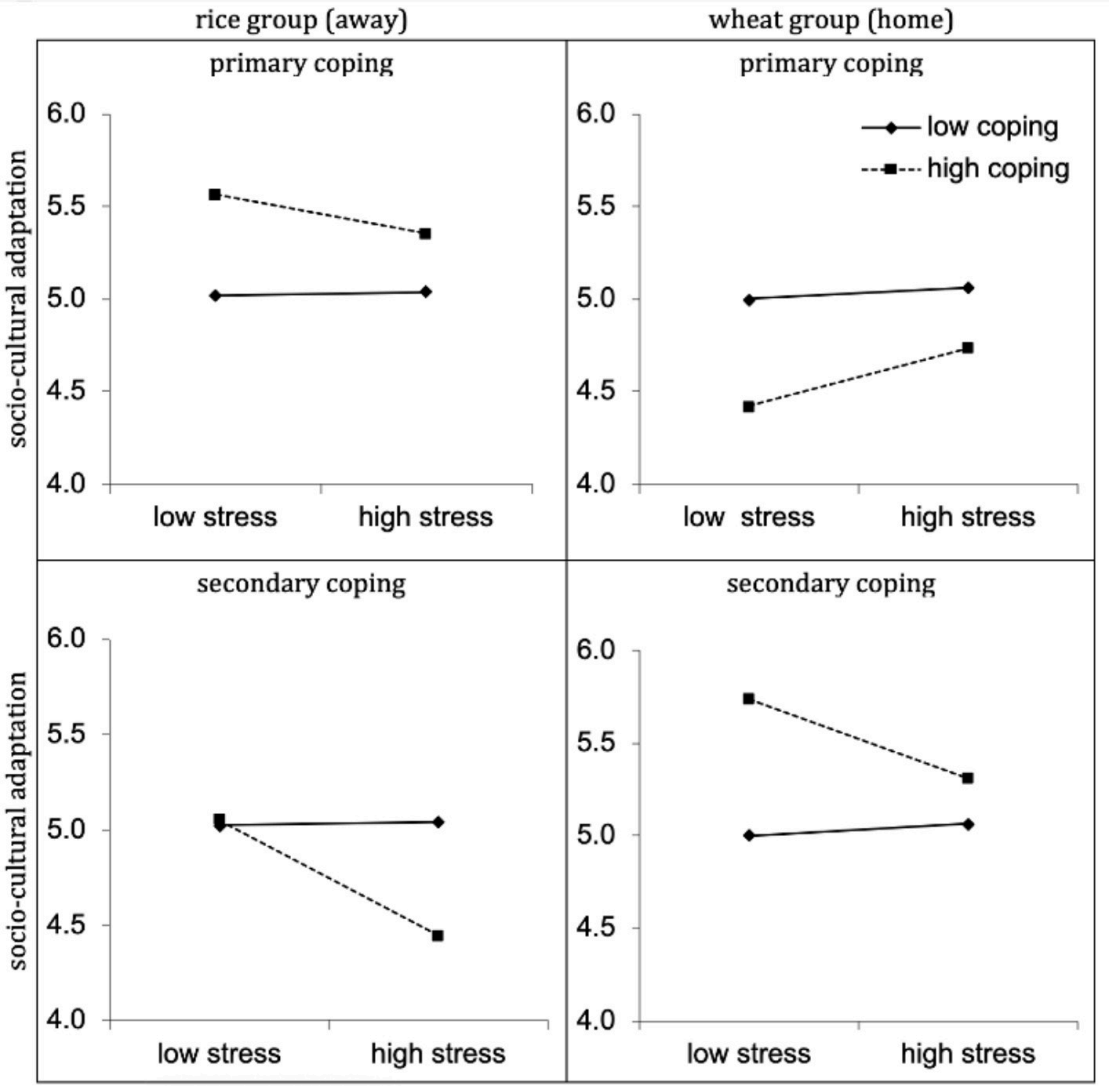
**Upper left panel shows how primary coping at time 1 functions across different levels of stress at time 1 on sociocultural adaptation at time 2 for rice participants in northern China.** Lower left panel shows that secondary coping alongside stress at time 1 was associated with decrements in sociocultural adaptation at time 2 for rice participants. Upper right and lower right depict no interaction effect for wheat (home) participants.

To decompose this interaction, two separate analyses for the rice and wheat group were conducted (Table 2). The interaction of stress by secondary coping was significant for the rice group (*β* = -.28, *p* = .036), but not for the wheat group (*β* = .14, *p* = .460). Simple slope analyses were performed using ModGraph. These analyses showed that under conditions of high initial stress, secondary coping exacerbated the negative effects of acculturative stress on sociocultural adaptation (see bottom left panel of Fig 2). Specifically, when the ‘away’ participants used high levels of secondary coping, stress was associated with decrements in sociocultural adaptation time 2; *t*(96) = -1.96, *p* = .051, Cohen’s *d* = .40. Under low levels of stress, it made no difference if they used secondary coping or not *t(*96) < 1.

**Table 2:** Regression analyses of stress, and coping predictors on sociocultural adaptation by origin (Study 1). Analyses for the rice group are presented on the left, analyses for the wheat group on the right.

	rice group (away)						wheat group (home)					
	step 1		step 2		step 3		step 1		step 2		step 3	
	beta	p	beta	p	beta	p	beta	p	beta	p	beta	p
cultural distance (t1)	-.17	.08	-.15	.14	-.12	.21	-.41	.00	-.35	.00	-.35	.00
living time	.09	.38	.09	.38	.09	.36	.00	.99	.02	.86	.02	.80
first time	-	-	-	-	-	-	.01	.89	.04	.65	.03	.75
stress (t1)			-.14	.16	.00	.98			-.08	.42	.07	.56
primary coping												
primary coping (t1)			-.04	.72	-.01	.96			-.27	.04	-.40	.00
stress x primary					-.03	.84					.21	.27
secondary coping												
secondary coping (t1)			.14	.20	.16	.14			.41	.00	.47	.00
stress x secondary					-.28	.04					.14	.46
model statistics												
F (df)	1.98 (2, 101)		1.55 (5, 98)		2.20* (7, 96)		5.54** (3, 81)		5.07*** (6,78)		5.13*** (8, 76)	
R^2^	.04		.07		.14		.17		.28		.35	
ΔF (df)			1.25 (3, 98)		3.64* (2, 96)				3.98* (3, 78)		4.10* (2, 76)	
ΔR^2^			.04		.07				.11		.07	

The variable first time was omitted for the rice group analyses, as all participants had left their hometown.

## Discussion

At the independent wheat site, endorsing secondary coping was maladaptive for the rice group, leading to lower sociocultural adaptation. The wheat group showed no interaction effects of coping with stress. Taken together, these results point at culturally normative differences in coping style. However, the results could also be explained by a certain ‘insider-outsider’ effect. As the outsider, the rice group may simply need to utilize more primary coping as they struggle to adapt to more cultural differences.

In order to fully understand effect of coping across the wheat-rice border, Study 2 replicates the study in the rice area (southern China). If the findings of Study 1 are due to an insider-outsider difference, we should find the same pattern with students who move from the wheat area to the rice area. However, if the coping congruency explanation is correct, we should find the opposite pattern—that secondary coping works better for new arrivals in the rice area.

## Study 2

In Study 2, we sought to replicate findings and expand on the results from Study 1. We postulated that primary coping culture and secondary coping culture vary between the rice and wheat regions. As people cross this border, the most culturally congruent coping strategy changes. We used another hierarchical linear regression analysis to test our prediction that among the wheat ‘away group,’ those who use primary coping will demonstrate worse sociocultural adaptation. Simultaneously, we anticipated that secondary coping would be more effective in reducing the negative impacts of stress on sociocultural adaptation. To test our predictions on the utility of coping strategies, we expected to observe a variation in coping (primary vs. secondary) based on origin of group.

## Methodology

### Participants & procedure

**Fig 3:**
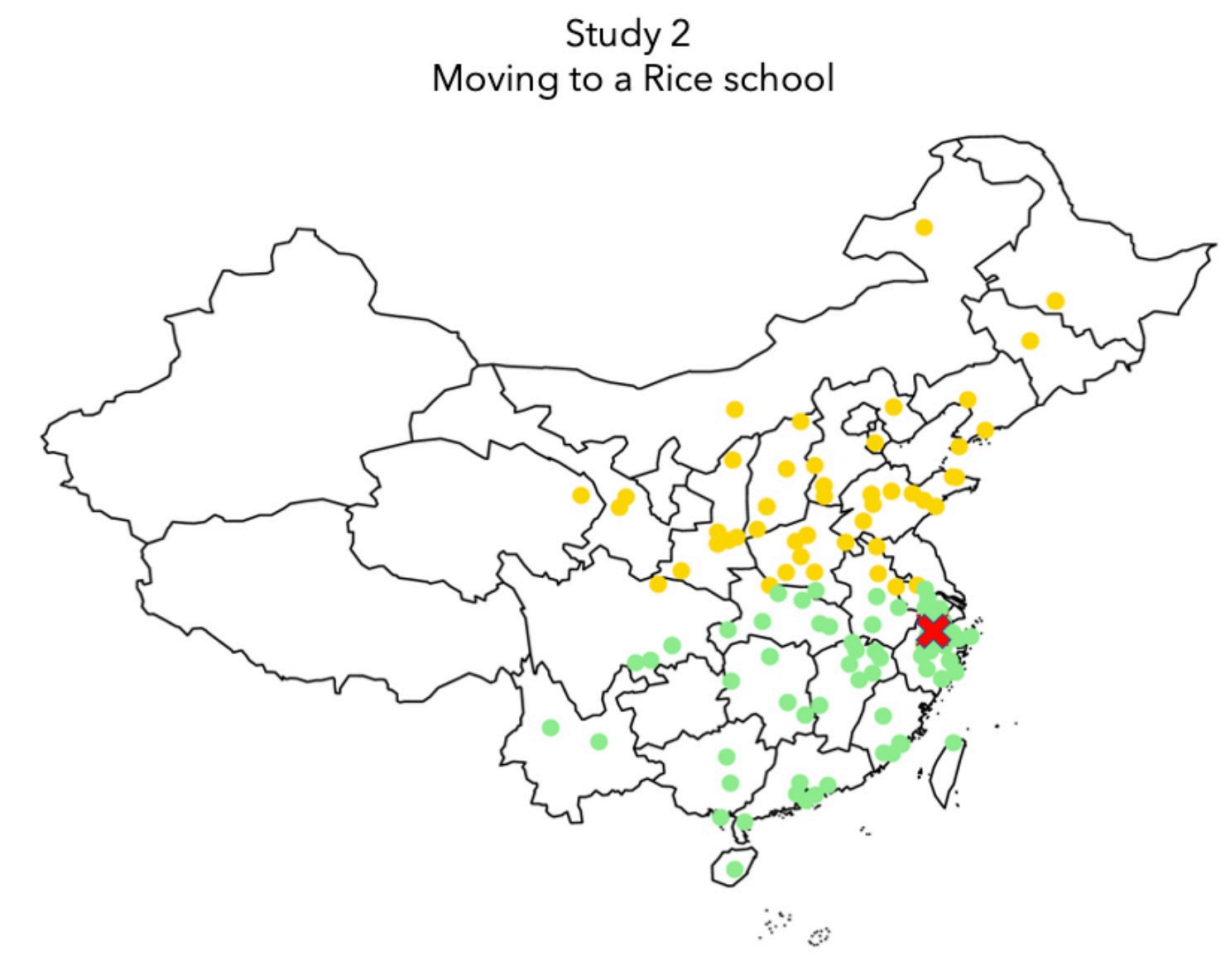
Location of student’s hometown in Study 2. Participants’ origin was recorded based on their hometown. Yellow dots represent where students from wheat areas originated from. Green dots represent where students from rice areas originated from. Test site was a top-tier university in Hangzhou.

Longitudinal data was collected from a university in southeastern China. Participants were from around China (see Fig 3). The study followed the same procedures as Study 1. During the data collection, participants were free to withdraw from the study at any time, and participation in the study did not impact their grade or standing in the school.

### Materials

The same materials were used in Study 2 as in Study 1. Reliability was good for perceived cultural distance (α > .90), perceived stress (α > .80), primary coping (α > .80), secondary coping (α > .75), and sociocultural adaptation (α > .90).

### Preliminary analyses

In total, *n =* 80 newcomers were from the northern wheat region and *n* = 113 came from the south rice region. In addition, 45% of the wheat region group came from a city, while in the rice (home) group, 38% were from a city. Gender did not significantly differ, and females made up 40% of the respective group clusters. A substantial difference emerged regarding the amount of time spent living in the host city of the university—the wheat group reported being in the city an average of 55 days, while the rice group had been in the city on average 330 days (*t* (183) *=* 6.01, *p =* .001). Additionally, the wheat group was, on average, about 9 months (20.1 years of age) older than those from the rice region (19.33) (*t* (183) *= 3*.*89*, *p* = .001*)*. A post-hoc power analysis was conducted and found this sample size has .93 to detect a medium effect size.

In total, 193 participants completed both time waves, for a response rate of 73%. Attrition analyses were conducted to determine if there was any sample bias due to the loss of participants in measurement 1. Analyses indicated one significant difference: primary coping (*t* = -3.10, *p* = .002). This suggests that those who dropped out of the study (n = 70) reported less primary coping (*M* = 3.16 *SD* = .49 vs. *M* = 3.33 *SD* = .44). All other testable variables showed no significant differences (Age *t* < 1; origin *t* < 1; gender *t* < 1; PCD t < 1; secondary coping *t* < 1; stress *t* < 1; living time in the city *t* < 1).

## Results

### Longitudinal moderation analysis

To examine whether the relationship between stress and adaptation was moderated by variations in coping and student geographical origin, a four-step hierarchical regression was conducted (Table 3). In the first step, sociocultural adaptation was regressed on control variables. The second step added stress (t1), primary coping (t1), secondary coping (t1), and students’ rice-wheat origin.

**Table 3:** Regression analyses of stress, origin, and coping predictors on sociocultural adaptation (Study 2).

	step 1 basic model		step 2 main effects		step 3 2-way interactions		step 4 3-way interactions	
	beta	p	beta	p	beta	p	beta	p
cultural distance (t1)	-.44	.00	-.38	.00	-.40	.00	-.42	.00
living time	.16	.02	.21	.00	.20	.00	.21	.00
first time	-.05	.46	-.06	.39	-.06	.41	-.11	.12
stress (t1)			-.06	.42	.02	.87	.02	.82
origin: rice vs wheat			-.05	.51	-.03	.69	-.03	.74
stress x origin					-.11	.21	-.12	.18
primary coping								
primary coping (t1)			.13	.15	.14	.21	.18	.11
stress x primary					-.24	.01	-.06	.62
origin x primary					-.15	.16	-.25	.03
stress x origin x primary							-.28	.03
secondary coping								
secondary coping (t1)			.14	.09	.06	.61	.04	.69
stress x secondary					.25	.01	.05	.70
origin x secondary					.20	.06	.25	.02
stress x origin x secondary							.31	.02
model statistics								
F (df)	15.41*** (3,169)		9.19*** (7, 165)		6.78 ***(12, 160)		6.46*** (14,158)	
R^2^	.22		.28		.34		.36	
ΔF (df)			3.77** (4,165)		2.73* (5,160)		3.33* (2,158)	
ΔR^2^			.07		.06		.03	

Categorical Variable: First time to leave home town (1 = yes; 0 = No)

* *p* <. 05

** *p* < .01

*** *p* < .001

Secondary coping was marginally associated with higher adaptation (*β* = .15, *p* < .09, *β* = .14, *p* = .091). The addition of the two-way interactions in the third step did significantly improve the model, accounting for 6% change in variance, Δ*F*(5, 160) = 2.73. Interactions between (1) stress and primary coping and (2) stress and secondary coping were both significant (*β* = -.24, *p* = .012 and *β* = .25, *p* = .007). A simple slope analysis of stress by primary coping interaction yielded a significant interaction at high levels of primary coping, *t* (173) = - 2.50, p = .014; suggesting that high levels of primary coping exacerbates stress sociocultural adaptation, leading to worse adjustment at Time 2. In contrast, a simple slope analysis of the stress by secondary coping interaction revealed that secondary coping buffered stress, leading to better sociocultural adaptation *t*(173) = 3.022, p = .002. There was also a (marginally) significant interaction between secondary coping and origin (*β* = .20, *p* = .061).

The four-step model explained 36% of the variance in sociocultural adaptation at time 2, *F*(14, 158) = 6.46, *p <* .001. In the full model (step-four), the three-way interactions explained 3% of the unique variance of sociocultural adaptation time 2; *F*(2, 158) = 3.33, p = .038. Results indicated a main effect for cultural distance at time 1, (*β =* -.42, *p* < .001), and living time (*β =* .21, *p* < .01). In addition, there was a significant interaction between rice-wheat origin and primary coping (*β* = -.25, *p* = .028), as well as between rice-wheat origin and secondary coping (*β =* .25, *p* = .021). The simple slope analysis for primary coping by rice-wheat origin did not yield any significant results; rice ‘home’ group: *t*(173) = 1.60, *ns*, and wheat ‘away’ group: *t*(173) = 1.55, *ns*. However, the simple slope analysis for the interaction between secondary coping and rice-wheat origin indicated that for the wheat ‘away’ group, secondary coping at time 1 was positively related with sociocultural adaptation at time 2; *t*(173) = 3.34, *p* = .001, Cohen’s *d =* .51. Simple slope analysis did not yield any significance for the rice ‘away group; *t*(173) = .395, *ns*.

Finally, in step four, both 3-way interactions were significant: stress x origin x primary (*β* = -.28, *p* = .026), and stress x origin x secondary (*β =* .31, *p* = .015). Additional analyses were implemented by origin separately, and results indicated that the interactions (stress x coping type) were present in the wheat ‘away’ group only. Specifically, for wheat participants, there was an interaction between stress and primary coping (*β = -*.43, *p* = .005).

Simple slope analyses showed that, for wheat participants, primary coping at time 1 amplified the negative effects of stress on sociocultural adaptation time 2 (Table 4). For people who experienced high stress, primary coping was negatively related to sociocultural adaptation at time 2; *t*(73) high = -3.07, *p* = .003, Cohen’s *d =* .72. On the other hand, for people who did not experience stress, primary coping made little difference. When low levels of primary coping were used, stress was not significantly related with sociocultural adaptation time 2, *t*(73) = -1.35, *ns*.

**Table 4:** Regression analyses of stress, and coping predictors on sociocultural adaptation by origin (Study 2). Analyses for the wheat group are presented on the left, analyses for the rice group on the right.

	wheat group (away)						rice group (home)					
	step 1		step 2		step 3		step 1		step 2		step 3	
	beta	p	beta	p	beta	p	beta	p	beta	p	beta	p
cultural distance (t1)	-.31	.01	-.23	.04	-.31	.01	-.51	.00	-.47	.00	-.47	.00
living time	.02	.90	.07	.55	.05	.63	.23	.01	.29	.00	.29	.00
first time	-	-	-	-	-	-	-.21	.02	-.20	.03	-.19	.04
stress (t1)			-.14	.22	-.14	.18			.01	.90	.02	.86
primary coping												
primary coping (t1)			.03	.83	-.21	.15			.20	.10	.19	.14
stress x primary					-.43	.01					-.05	.67
secondary coping												
secondary coping (t1)			.30	.03	.43	.00			.05	.65	.06	.60
stress x secondary					.44	.00					.03	.81
model statistics												
ΔF (df)	3.75* (2, 70)		4.07** (5, 67)		4.88*** (7, 65)		13.15*** (3, 97)		7.93*** (6, 94)		5.86*** (8, 92)	
R^2^	.10		.23		.35		.29		.34		.34	
ΔF (df)			3.97* (3, 67)		5.53** (2, 65)				2.22 (3, 94)		.10 (2, 92)	
ΔR^2^			.14		.11				.05		.00	

The variable first time was omitted for the wheat group analyses, as all participants had left their hometown.

* *p* <. 05

** *p* < .01

*** *p* < .001

In contrast to the moderation effect of primary coping, a significant positive interaction between stress and secondary coping (*β =* .44, *p* = .002) was observed among participants from wheat provinces. Secondary coping buffered the negative effects of stress on sociocultural adaptation at Time 2. Among people who experienced high stress, secondary coping stress was related to better sociocultural adaptation at Time 2; *t*(73) = 1.93, *p* = .058, Cohen’s *d* = .45. However, among people who experienced no stress, secondary coping was not related with sociocultural adaptation time 2; *t*(73) = -1.45, *ns*.

### Discussion

Study 2 found the opposite results from Study 1. In the rice region (southern China), primary coping was maladaptive for people who had arrived from the wheat region. People who used primary coping had worse adaptation at Time 2. In contrast, secondary coping buffered the negative effects of stress on sociocultural adaptation for the arrivals from the wheat region. Thus, for north-to-south migration, we found that secondary coping was a beneficial moderator in the adaptation process. As the pattern was the converse of Study 1, this suggests that the most successful coping style depends on cultural region. Additionally, it is further supported that the pattern in Study 1 is best explained by cultural coherence, and is not simply a manifestation of the “outsider effect.”

## General discussion

Across the globe, different ecologies craft different cultures. Overall, the findings of these two studies demonstrate that the best coping mechanism for those who move from one cultural region to another is that which is congruent with their new environment, rather than their home culture. In particular, this study tested whether the most adaptive coping style differs between China’s two agricultural regions, and the results show that educational migrants between these regions would do well to adapt their coping styles to those of their host region.

Our findings first lend further support to the contention that important cultural differences do exist among China’s massive 1.4 billion population. Past research has demonstrated certain key differences based on agriculture—that China’s rice and wheat cultural regions are in some ways significantly different. This study expands on this, showing that normalized coping styles vary between these two regions—.the wheat region in northern China seems to reward primary coping styles, while southern China’s rice culture seems to reward secondary coping styles. Thus, those who travel from one region to the other benefit more by adopting the prevailing coping mechanism of that region.

Additionally, we found evidence that the effects of coping style—either positive or negative—depended on stress levels. For people who experienced very little stress after moving to the new region, their choice of coping style did not matter. Conversely, among people who experienced high levels of stress, those who adopted the coping style which was congruent with their host region adapted more successfully to that culture.

There is some speculative observational data suggesting that primary coping is more common in northern China that complements our findings. To test for regional differences, researchers moved chairs in Starbucks so that they were partially blocking the aisle [37]. Researchers then observed whether people moved the chair out of the way or adjust their body to fit through the chairs. Actively moving the chair plausibly maps onto primary coping because it involves actively changing the environment, whereas adjusting the body to fit through the chairs plausibly maps onto secondary coping because it involves fitting into the environment. People in the wheat region were more likely to move the chair than people in the rice region, suggesting that primary coping is more common in the wheat region. Chair moving was also correlated with regional differences in self-inflation on the sociogram task, as measured in a previous study [4]. This suggests that there is a link between individualism and primary coping in China.

### Reasons for such differences

**Fig 4:**
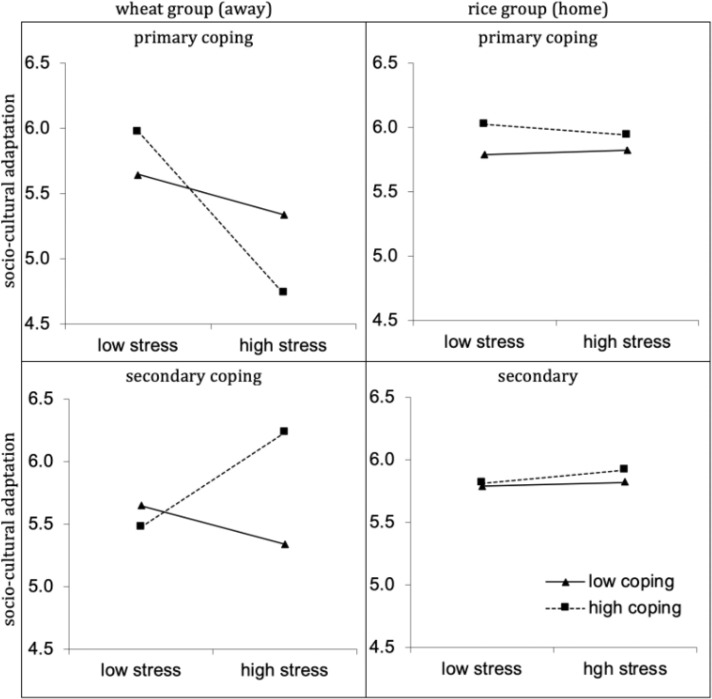
**Upper left panel shows how primary coping at time 1 exacerbated the negative effects of stress at time 1 on sociocultural adaptation at time 2 for wheat participants in southern China.** Lower left panels show how secondary coping at time 1 buffers the negative effects of stress time 1 on sociocultural adaptation time 2 for wheat participants in southern China. Upper right and lower right depict no interaction effect for rice (home participants).

Perhaps the best explanation of these results is rooted in agriculture. According to Talhelm and colleagues [4], rice cultivation requires extensive coordination and sharing of resources, which fosters reciprocal obligations and tight relationships among close social networks. In these regions, the cultural norms of context require individuals to navigate the interconnected web of relationships [38]. These social ties create a context where collectivist ways of life become normalized—where the needs of the community are placed over the needs of the individual. In such a social environment with tight social ties and a strong collective identity, it is likely that secondary coping would become normalized. Thus, it is not surprising that newly arrived migrants from wheat cultures in the north of China struggle to utilize their primary coping style to deal with the challenges of the new cultural context. However, if they are able to deploy secondary coping in response to high stress, it improves their adaptation process. This is displayed in Fig 4.

The same agricultural roots can explain well the findings in Study 1. Given the association of wheat cultures with analytic thought and independent farming styles, primary coping is more likely to become the normalized coping method in these regions [4, 37]. Thus, migrants from south to north will more easily adapt to their new environment if they adopting primary coping to a greater degree. However, this does not overwhelm China’s general cultural predilection, as an interdependent, collectivistic culture, for secondary coping—a trend also observed in our results.

In order to set these results in proper perspective, a few further observations must be made. First, it is important to note that Yamaguchi [39] ascertained that East Asian cultures do not use primary coping less than in the West; rather, due to the tight knit norms, social harmony and face saving are unavoidable elements in collective cultures [40, 41]. Thus, studies which seek to measure secondary coping levels in East Asian cultures are perhaps hitting on a false positive—it may not be that East Asian cultures ‘prefer’ secondary coping, but rather that it is unknowingly adopted out of social necessity. Second, on the links from rice and wheat to cultural differences, recent research in India has found differences in cognitive and social consequences between dry upland rice farming vs. rice paddy farming [11]. Overall, this supports the theory that environments shape cultural norms. However, most, if not all, of China’s rice production takes the form of irrigated paddy farming, where villagers actively irrigated, shared water, and harvested the season’s crop together [38]. Thus, the cultural links to different styles of rice-farming in India may not be found in China.

This paper contributes to existing literature in several ways. First, it addresses and provides support for the existence of regional, cultural differences within China [42, 2]. Second, this paper contributes to acculturation literature by longitudinally examining the acculturative changes of migrants within a single nation. Third, it contributes to the cultural-ecological literature by observing a theoretical link between ecological roots of culture (subsistence theory) and observed patterns in culturally normalized coping styles.

While our research does extend the literature by linking culture, ecology and acculturation, within the stress-coping paradigm, we do recognize one major shortcoming. In this study, we failed to measure two mediating variables, namely, interdependence/ interdependence, and cultural tightness/looseness [3, 9]. As postulated by the ecocultural framework, these variables are established pathways that link subsistence practices to psychological outcomes [2]—i.e., the subsistence method (rice or wheat) created either a tight, interdependent or loose, independent culture, which in turn normalized either secondary or primary coping styles. In absence of directly studying these links, we note this limitation, yet a recent study did establish a link between rice farming and tighter social norms in China and worldwide [43]. We hope future researchers can investigate how norm tightness is linked with coping styles during stressful situations.

In conclusion, besides its contributions to scholarly research, our study offers practical support to the hundreds of millions of internal migrants in China, encouraging them to employ ‘culturally fit’ coping strategies to facilitate their adaptation in their specific eco-cultural environment. It also provides expansive research a migrant group in China that deserves further attention—educational migrants [13, 44, 45]. As the Chinese proverb suggests, “Enter village, follow custom” (入乡随俗), we hope this research provides more evidence of how coping styles can be seen as social norms and behaviors within a given community, and how sojourners may be encouraged to ‘adapt’ their very methods of dealing with stress while adapting to the new and unknown.

## References

[1] DESA U. United Nations Department of Economic and Social Affairs/Population Division (2009b): World Population Prospects: The 2008 Revision. Internet: http://esa. un. org/unpp (gelesen am 16. 2010 Sep.

[2] Berry JW. Ecocultural perspective on human behaviour. Uskul A., K. & Oishi S.(Ed.), Socio-Economic Environment and Human Psychology. 2018:3–2.

[3] Harrington JR, Gelfand MJ. Tightness–looseness across the 50 united states. Proceedings of the National Academy of Sciences. 2014 6 3;111(22):7990–5. 10.1073/pnas.1317937111 PMC4050535 24843116

[4] Talhelm T, Zhang X, Oishi S, Shimin C, Duan D, Lan X, et al Large-scale psychological differences within China explained by rice versus wheat agriculture. Science. 2014 5 9;344(6184):603–8. 10.1126/science.1246850 24812395

[5] Berry JW. Human ecology and cognitive style: Comparative studies in cultural and psychological adaptation. John Wiley & Sons; 1976.

[6] Berry JW, Bahuchet S, Van De Koppel J, Annis R, Senechal C, Cavalli-Sforza LL, et al On the edge of the forest. Cultural adaptation and cognitive development in Central Africa. 1986.

[7] Schug J, Yuki M, Maddux W. Relational mobility explains between-and within-culture differences in self-disclosure to close friends. Psychological Science. 2010 10;21(10):1471–8. 10.1177/0956797610382786 20817913

[8] Bach RA, Defever AM, Chopik WJ, Konrath SH. Geographic variation in empathy: A state-level analysis. Journal of research in personality. 2017 6 1;68:124–30.

[9] Uskul AK, Kitayama S, Nisbett RE. Ecocultural basis of cognition: Farmers and fishermen are more holistic than herders. Proceedings of the National Academy of Sciences. 2008 6 24;105(25):8552–6. 10.1073/pnas.0803874105 PMC2438425 18552175

[10] Mishra RC, Sinha D, Berry JW. Ecology, acculturation and psychological adaptation: A study of adivasis in Bihar. Sage Publications, Inc; 1996.

[11] Mishra RC, Berry JW. Ecology, culture and human development: Lessons for Adivasi education. SAGE Publishing India; 2017 10 30.

[12] Wang L, Mesman J. Child development in the face of rural-to-urban migration in China: A meta-analytic review. Perspectives on Psychological Science. 2015 11;10(6):813–31. 10.1177/1745691615600145 26581737

[13] Gui Y, Berry JW, Zheng Y. Migrant worker acculturation in China. International Journal of Intercultural Relations. 2012 7 1;36(4):598–610.

[14] Littrell RF, Alon I, Wai Chan K. Regional differences in managerial leader behaviour preferences in China. Cross Cultural Management: An International Journal. 2012 7 27;19(3):315–35.

[15] Van de Vliert E, Yang H, Wang Y, Ren XP. Climato-economic imprints on Chinese collectivism. Journal of Cross-Cultural Psychology. 2013 5;44(4):589–605.

[16] Kulich SJ, Zhang R. The multiple frames of “Chinese” values: From tradition to modernity and beyond. The Oxford handbook of Chinese psychology. 2010:241–78.

[17] Ward C. The A, B, Cs of acculturation. The handbook of culture and psychology. 2001 Sep 20:411–45.

[18] Geeraert N, Li R, Ward C, Gelfand M, Demes KA. A tight spot: How personality moderates the impact of social norms on sojourner adaptation. Psychological science. 2019 3;30(3):333–42. 10.1177/0956797618815488 30673368 PMC6419235

[19] Sam DL, Berry JW, editors. The Cambridge handbook of acculturation psychology. Cambridge University Press; 2006 8 3.

[20] Lazarus RS, Folkman S. Stress, appraisal, and coping. Springer publishing company; 1984 3 15.

[21] Lazarus RS, Lazarus RS. Emotion and adaptation. Oxford University Press on Demand; 1991.

[22] Berry J.W. Stress perspectives on acculturation. In Cambridge handbook of acculturation psychology. Cambridge: Cambridge University Press, 2006, 43–57.

[23] Heppner PP, Wei M, Neville HA, Kanagui-Muñoz M. A cultural and contextual model of coping.

[24] Morling B, Evered S. Secondary control reviewed and defined. Psychological bulletin. 2006 3;132(2):269 10.1037/0033-2909.132.2.269 16536644

[25] Rothbaum F, Weisz JR, Snyder SS. Changing the world and changing the self: A two-process model of perceived control. Journal of personality and social psychology. 1982 1;42(1):5.

[26] Skinner EA, Zimmer-Gembeck MJ. The development of coping. Annu. Rev. Psychol. 2007 1 10;58:119–44. 10.1146/annurev.psych.58.110405.085705 16903804

[27] Kitayama S., Duffy S., & Uchida Y. (2007). Self as cultural mode of being. Kitayama S, Duffy S, Uchida Y. Self as cultural mode of being. Handbook of cultural psychology. 2007:136–74.

[28] Wei M, Liao KY, Heppner PP, Chao RC, Ku TY. Forbearance coping, identification with heritage culture, acculturative stress, and psychological distress among Chinese international students. Journal of counseling psychology. 2012 1;59(1):97 10.1037/a0025473 21928876

[29] Szabo A, English AS, Zhijia Z, Jose P, Ward C, Jianhong M. Is the utility of secondary coping a function of ethnicity or the context of reception? A longitudinal study across Western and Eastern cultures. Journal of Cross-Cultural Psychology. 2017 9;48(8):1230–46.

[30] Rothbaum F, Morelli G, Rusk N. Attachment, learning, and coping. Advances in culture and psychology. 2011:153–215.

[31] English AS, Zeng ZJ, Ma JH. The stress of studying in China: primary and secondary coping interaction effects. SpringerPlus. 2015 12 1;4(1):755. 26693113 10.1186/s40064-015-1540-3 PMC4666850

[32] Szabo A, Ward C, Jose PE. Uprooting stress, coping, and anxiety: A longitudinal study of international students. International Journal of stress management. 2016 5;23(2):190.

[33] Demes KA, Geeraert N. Measures matter: Scales for adaptation, cultural distance, and acculturation orientation revisited. Journal of Cross-Cultural Psychology. 2014 1;45(1):91–109.

[34] Cohen S, Kamarck T, Mermelstein R. A global measure of perceived stress. Journal of health and social behavior. 1983 12 1:385–96. 6668417

[35] Carver CS, Scheier MF, Weintraub JK. Assessing coping strategies: a theoretically based approach. Journal of personality and social psychology. 1989 2;56(2):267 10.1037//0022-3514.56.2.267 2926629

[36] Dong X, Talhelm T, Ren X. Teens in Rice County Are More Interdependent and Think More Holistically Than Nearby Wheat County. Social Psychological and Personality Science. 2018:1948550618808868.

[37] Talhelm T, Zhang X, Oishi S. Moving chairs in Starbucks: Observational studies find rice-wheat cultural differences in daily life in China. Science advances. 2018 4 1;4(4):eaap8469 10.1126/sciadv.aap8469 29707634 PMC5916507

[38] Talhelm T, Oishi S. How Rice Farming Shaped Culture in Southern China. In Socio-Economic Environment and Human Psychology: Social, Ecological, and Cultural Perspectives. Oxford, Oxford University Press, 2018 2 28:53.

[39] Yamaguchi S. Culture and control orientations. The handbook of culture and psychology. 2001 9 20:223–43.

[40] Fung HH, Ng SK. Age differences in the sixth personality factor: Age differences in interpersonal relatedness among Canadians and Hong Kong Chinese. Psychology and Aging. 2006 12;21(4):810 10.1037/0882-7974.21.4.810 17201500

[41] Heine SJ, Hamamura T. In search of East Asian self-enhancement. Personality and Social Psychology Review. 2007 2;11(1):4–27. 10.1177/1088868306294587 18453453

[42] Van de Vliert E. Climato-economic habitats support patterns of human needs, stresses, and freedoms. Behavioral and Brain Sciences. 2013 10;36(5):465–80. 10.1017/S0140525X12002828 23985270

[43] Talhelm T., & English A. S., Historically rice-farming societies have tighter social norms in China and worldwide. Manuscript under review at Proceedings of National Academy of Sciences (2020). 10.1073/pnas.1909909117 PMC7443949 32732432

[44] English AS, Worlton DS. Coping with uprooting stress during domestic educational migration in China. Journal of Pacific Rim Psychology. 2017;11.

[45] English AS, Kunst JR, Sam DL. Climatic effects on the sociocultural and psychological adaptation of migrants within China: A longitudinal test of two competing perspectives. Asian Journal of Social Psychology. 2019 9;22(3):244–55.

